# Torsed Riedel’s lobe

**DOI:** 10.1007/s00247-026-06586-2

**Published:** 2026-04-07

**Authors:** Lucas Castro, Ivna Moura, Bruno Silva, Carolina Borges, Silvio Albuquerque, Eduardo Silva

**Affiliations:** Instituto de Medicina Integral Professor Fernando Figueira, Recife, Brazil


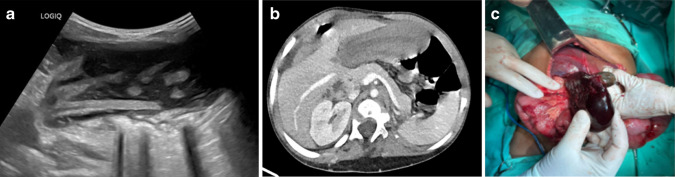


A 4-year-old boy was admitted with one day history of abdominal pain and fever. The patient had multiple co-morbidities (lipomyelomeningocele, cloacal exstrophy, omphalocele, urethral stenosis, multicystic left kidney, anorectal anomaly, and congenital clubfoot). **a** Transverse ultrasound image showed a hypoechoic lesion adjacent to the liver, with linear echogenic bands. **b** Transverse contrast enhanced computed tomography image showed a heterogeneous mass with hyperattenuating areas suggestive of hemorrhage, in contact with the right hepatic lobe and transverse colon. **c** Intraoperative findings confirmed torsion of a Riedel’s hepatic lobe. Torsion of an accessory hepatic lobe is a rare but known cause of an acute abdomen. The presence of a hypoechoic lesion adjacent to the liver, containing echogenic internal bands, should prompt radiologists to consider the diagnosis of accessory hepatic lobe torsion.

## Data Availability

No datasets were generated or analysed during the current study.

